# Effect of dexamethasone as osteogenic supplementation in in vitro osteogenic differentiation of stem cells from human exfoliated deciduous teeth

**DOI:** 10.1007/s10856-020-06475-6

**Published:** 2021-01-19

**Authors:** Mariane Beatriz Sordi, Raissa Borges Curtarelli, Izabella Thaís da Silva, Gislaine Fongaro, Cesar Augusto Magalhães Benfatti, Ricardo de Souza Magini, Ariadne Cristiane Cabral da Cruz

**Affiliations:** 1 Center for Research on Dental Implants, Federal University of Santa Catarina, Delfino Conti Street, Florianópolis, 88040-900 Brazil; 2 Laboratory of Applied Virology, Federal University of Santa Catarina, Henrique da Silva Fontes Avenue, Florianópolis, 88040-900 Brazil; 3 Department of Pharmaceutics Science, Federal University of Santa Catarina, Delfino Conti Street, Florianópolis, 88040-900 Brazil; 4 Department of Microbiology, Immunology, and Parasitology, Federal University of Santa Catarina, Henrique da Silva Fontes Avenue, Florianópolis, 88040-900 Brazil; 5 Department of Dentistry, Federal University of Santa Catarina, Delfino Conti Street, Florianópolis, 88040-900 Brazil

## Abstract

In in vitro culture systems, dexamethasone (DEX) has been applied with ascorbic acid (ASC) and β-glycerophosphate (βGLY) as culture media supplementation to induce osteogenic differentiation of mesenchymal stem cells. However, there are some inconsistencies regarding the role of DEX as osteogenic media supplementation. Therefore, this study verified the influence of DEX culture media supplementation on the osteogenic differentiation, especially the capacity to mineralize the extracellular matrix of stem cells from human exfoliated deciduous teeth (SHED). Five groups were established: G1—SHED + Dulbecco’s Modified Eagles’ Medium (DMEM) + fetal bovine serum (FBS); G2—SHED + DMEM + FBS + DEX; G3—SHED + DMEM + FBS + ASC + βGLY; G4—SHED + DMEM + FBS + ASC + βGLY + DEX; G5—MC3T3-E1 + α Minimal Essential Medium (MEM) + FBS + ASC + βGLY. DNA content, alkaline phosphatase (ALP) activity, free calcium quantification in the extracellular medium, and extracellular matrix mineralization quantification through staining with von Kossa, alizarin red, and tetracycline were performed on days 7 and 21. Osteogenic media supplemented with ASC and β-GLY demonstrated similar effects on SHED in the presence or absence of DEX for DNA content (day 21) and capacity to mineralize the extracellular matrix according to alizarin red and tetracycline quantifications (day 21). In addition, the presence of DEX in the osteogenic medium promoted less ALP activity (day 7) and extracellular matrix mineralization according to the von Kossa assay (day 21), and more free calcium quantification at extracellular medium (day 21). In summary, the presence of DEX in the osteogenic media supplementation did not interfere with SHED commitment into mineral matrix depositor cells. We suggest that DEX may be omitted from culture media supplementation for SHED osteogenic differentiation in vitro studies.

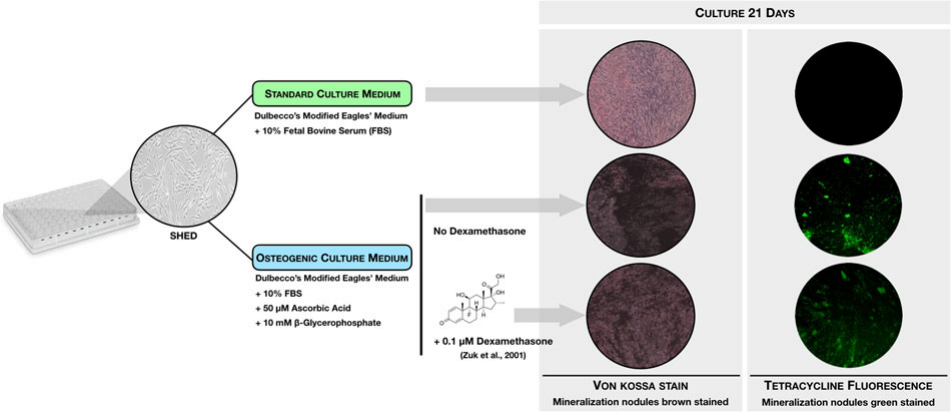

## Introduction

Researchers and clinicians are increasingly motivated to select mesenchymal stem cells (MSCs) from dental origins as a source of stem cells for tissue regeneration and cellular-based therapies [1–3]. Stem cells from human exfoliated deciduous teeth (SHED) are part of human dental tissues, corresponding to a subgroup of MSCs [4]. Therefore, SHED are a population of postnatal stem cells with the ability to differentiate into restrictive, but various cell types, including osteoblasts, chondroblasts, and neural cells. Since SHED are obtained from naturally exfoliated deciduous teeth, these cells stand as a unique, readily accessible, and noninvasive stem cell resource. Furthermore, SHED have been reported to exhibit high proliferation rates, the notable potential for differentiation, as well as mineralization of extracellular matrix capacity [5]. In addition, SHED exhibit distinct biological characteristics and gene expression profiles compared to other dental MSCs [3, 5–7].

Dexamethasone (DEX) is a steroid hormone and a potent synthetic member of the glucocorticoid family. Glucocorticoids are synthesized and released from the adrenal cortex, regulated by the hypothalamic–pituitary–adrenal axis, and actively act on several physiological functions. Interestingly, endogenous glucocorticoids are crucial for osteoblast differentiation during embryonic osteogenesis [8]. However, exposing embryos to elevated levels of glucocorticoids has been proved to lower the fetus birth weight and disorder the equilibrium of embryonic bone formation in vivo [9].

In in vitro culture systems, DEX has been applied along with ascorbic acid and β-glycerophosphate (βGLY) as media supplementation to induce MSCs to differentiate into osteoblasts. Several distinct effects on MSCs and osteoblasts have been reported. Therefore, DEX impact over MSCs seems to be related to the treatment duration, concentration, and stage of osteoblastic differentiation [10–13]. It has been demonstrated a positive stimulus of osteoblast lineage commitment in the presence of DEX at early stages of differentiation, but a downstream of bone matrix formation as osteoblastic differentiation progresses [12, 14, 15]. Rimando et al. [12] explained these findings by the glucocorticoid receptor (GR), since, in osteoblasts, GR is directly targeted by early (Runx2) and late (osteocalcin and bone sialoprotein) bone markers. GR positively regulates Runx2 while inhibits osteocalcin transcriptions. These results are in accordance with Lian et al. [14] that found inhibition of osteoblastic differentiation of MC3T3-E1 by the chronic use of DEX through the downregulation of osteocalcin gene expression. In addition, Wang et al. [15] suggested that a low dose of DEX favors MSCs proliferation and angiogenesis in vitro, and protects against apoptosis, while high concentrations of DEX decrease osteoblasts proliferation [10]. Conversely, Li et al. [16] found that MSCs under DEX treatment were more likely to differentiate into an adipocyte lineage rather than osteoblasts even under the induction of bone morphogenetic protein-2, thus DEX shifted the differentiation away from the osteoblast lineage.

The choice of the osteogenic supplementation medium for MSCs is crucial, considering that these cells with the respective medium will represent the positive control group in tissue engineering experiments and evaluation of the biomaterials and/or substances osteoinduction capacity [17]. In view of the inconsistency concerning the use of DEX as osteogenic media supplementation to differentiate MSCs, the present study aimed to verify the influence of DEX culture media supplementation on the DNA content and osteogenic differentiation, especially the capacity to mineralize the extracellular matrix of SHED. The null hypothesis was that the DEX would not influence the osteogenic culture media supplementation to DNA content and differentiation of SHED.

## Materials and methods

SHED were provided by the Curityba Biotech™ Cell Processing Center. These cells were collected from the pulp of naturally exfoliated deciduous teeth from three patients. Cells were characterized by the cell processing center before being sent to the laboratory. These cells were cultured in Dulbecco’s Modified Eagles’ Medium (DMEM; Gibco, Thermo Fisher Scientific) with 10% fetal bovine serum (FBS; Gibco, Thermo Fisher Scientific) (non-osteogenic DMEM) for subculturing at 37 °C with an atmosphere of 5% of CO_2_ until the passage number 6. For the experiments, the osteogenic media composition for SHED culture was different combinations of DMEM with 10% FBS, 50-µM L-ascorbic acid (ASC; A4544, Sigma-Aldrich), 10-mM βGLY (G9891, Sigma-Aldrich), and 0.1-µM DEX (D4902, Sigma-Aldrich) [18]. Tests were performed to determine DNA content and differentiation into an osteogenic-like lineage. Experimental control was performed using murine preosteoblasts MC3T3-E1 subclone 4 (American Type Culture Collection, CRL 2593) cultured with modified α-Minimum Eagles’ Medium (Gibco, Thermo Fisher Scientific), combining 10% FBS, 50-µM ASC, and 10-mM βGLY [19].

Five groups were established, namely: G1—SHED + DMEM + FBS; G2—SHED + DMEM + FBS + DEX; G3—SHED + DMEM + FBS + ASC + βGLY; G4—SHED + DMEM + FBS + ASC + βGLY + DEX; G5—MC3T3-E1 + αMEM + FBS + ASC + βGLY.

The sample size calculation for independent groups was performed using data from a pilot study, according to the equation [20]

$${n} = \left[ {\left( {{Sa}^{\mathrm{2}} + {Sb}^2} \right) \cdot \left[ {\frac{{\frac{{{Z}\alpha }}{2} + {Z}\beta }}{d}} \right]^2} \right]$$

where *n* = sample size per group; $\frac{{{Z}\alpha }}{2}$ = 1.96; *Zβ* = 0.84; *d* = minimum difference among mean; *Sa e Sb* = groups standard deviation.

$${n} = \left[ {\left( {1 + 0.51} \right) \cdot \left[ {\frac{{1.96 + 0.84}}{{1.51}}} \right]^2} \right]$$

*n* = 9.0298.

Considering that the sample size calculation indicated *n* = 9.0298 as a minimum sample for each group, the authors opted to use *n* = 12. Therefore, using 96-well plates, 2.0 × 10^4^ cells per well were seeded in quadruplicate and cultured at 37 °C and 5% CO_2_. Since the experiments were performed in triplicate, *n* = 12 for each evaluated group. Culture media were changed every 2–3 days. Experiments, aiming to evaluate DNA content and osteogenic differentiation, were performed at days 7 and 21, according to the methodologies described below.

### DNA content

DNA content was assessed through Quant-iT TM PicoGreen® dsDNA Reagent (P7589, Invitrogen, Thermo Fisher Scientific). This test acts by quantifying double-stranded DNA, even in small amounts in the solution, minimizing RNA and single-stranded DNA fluorescence detection. Analyses were performed according to the manufacturer’s recommendations, based on Cho et al. [21], with modifications. Readings were performed on a fluorescence spectrophotometer (SpectraMax M2e, Molecular Devices) at 360/440 nm (Ex/Em).

### Alkaline phosphatase (ALP) activity

To determine the ALP activity, the ALP Assay Fluorimetric Kit (Abnova) was used. Supernatants were collected and transferred to a 96-well plate following the manufacturer’s guidelines to perform fluorescence spectrophotometer readings (SpectraMax M2e, Molecular Devices) at 360/440 nm (Ex / Em). The results from ALP quantification were normalized to the DNA content.

### Calcium quantification in the extracellular medium

To quantify the free calcium content in the extracellular medium, the QuantiChrom^TM^ Calcium Assay Kit (BioAssay Systems) was used. Supernatants were collected and transferred to a 96-well plate following the manufacturer’s guidelines, and according to Cruz et al. [19]. Optical density readings were performed using a spectrophotometer (SpectraMax M2e, Molecular Devices) at 612 nm. The calcium quantification results were normalized to the DNA content.

### Extracellular matrix mineralization

The mineralization of the extracellular matrix was evaluated using three different methods.

Von Kossa staining was performed to visualize the mineralization nodules in the extracellular matrix. According to the method described by Cruz et al. [19], cells were fixed with 4% paraformaldehyde for 60 min and stained with 1% silver nitrate solution (Merck) for 30 min at room temperature, whilst protected from light. Counterstaining was performed with 0.1% eosin alcoholic solution (Sigma-Aldrich) for 10 min. Wells were photographed with a camera coupled to an inverted light microscope (Zeiss). Image J software (National Institute of Health) was employed to quantify the mineralization nodules.

Also for visualization and quantification of mineralization nodules in the extracellular matrix, fluorometric staining with tetracycline was performed. For this, all culture media from the five experimental groups were supplemented with 0.1% tetracycline hydrochloride (Sigma-Aldrich), and periodically changed. To perform the quantification, tetracycline-supplemented media were removed and replaced with standard culture media without tetracycline, according to the established protocol. Readings were performed on a fluorescence spectrophotometer (SpectraMax M2e, Molecular Devices) at 570 nm. Plates were taken to the inverted fluorescence microscope (Zeiss) to photograph the mineral nodules.

For quantification of extracellular matrix mineralization by alizarin red staining, cell fixation was performed with 4% paraformaldehyde for 15 min and stained with 3% alizarin red for 30 min. Staining was solubilized using 5% sodium dodecyl sulfate in 0.5-N HCl for 30 min. The solution was transferred to a new 96-well plate, and optical densities were analyzed using a spectrophotometer (SpectraMax M2e, Molecular Devices) at 490 nm, based on Pang et al. [22].

### Statistical analysis

Three independent experiments were performed to confirm the reproducibility of the results. Graph Pad Software Inc. (San Diego, CA, USA) was employed for statistical analysis. Results of DNA content, ALP activity, calcium quantification, and extracellular matrix mineralization quantification by von kossa, tetracycline, and alizarin red from all groups were compared through a one-way analysis of variance followed by the Tukey’s post-hoc test, comparing all treatments at each experimental time individually. Statistical analyses were also performed comparing the results of days 7 and 21 from each group through *t*-test. Differences between datasets with *p* < 0.05 were considered statistically significant.

## Results

### DNA content

**Fig. 1:**
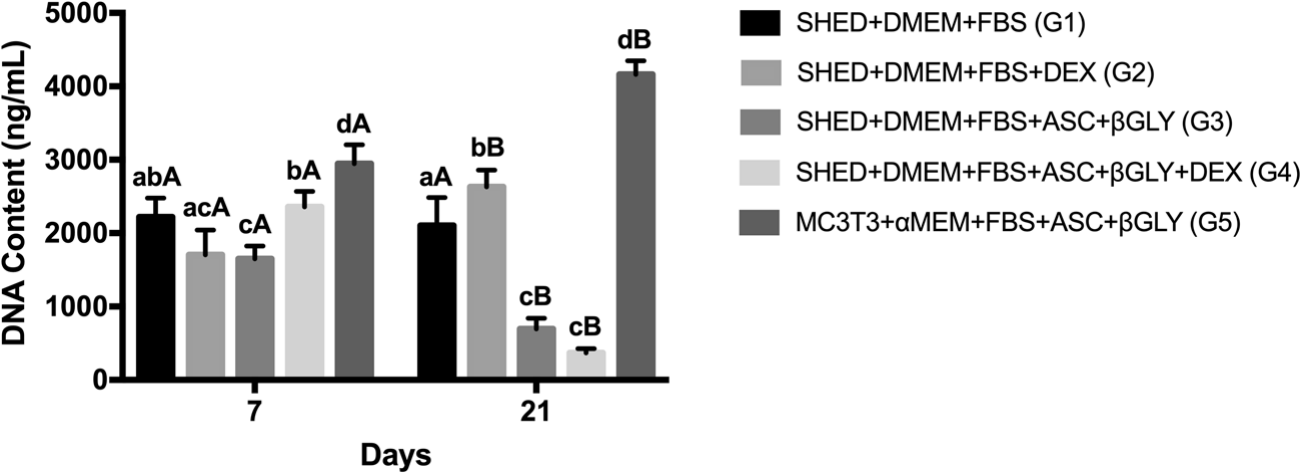
DNA content assay by PicoGreen® reagent on days 7 (*n* = 12 for each group) and 21 (*n* = 12 for each group). Different lower-case letters refer to a significant difference (ANOVA/Tukey test, *p* < 0.05) among groups at the same experimental time. Different capital letters indicate significant differences (Student test, *p* < 0.05) between the experimental times for the same group

DNA content assay results are shown in Fig. 1, where the main finding is that G4 demonstrated more DNA content than G3 (*p* = 0.0092) at day 7, while G3 and G4 demonstrated similar results on day 21 (*p* = 0.2909). An evident reduction in DNA content of G3 and G4 comparing day 7 with day 21 of the experiment (*p* = 0.0002 and *p* = 0.0001, respectively) was observed, while G1 maintained the DNA content (*p* = 0.6277), and G2 and G5 increased this quantification (*p* = 0.0053 and *p* = 0.0004, respectively).

### ALP activity

**Fig. 2:**
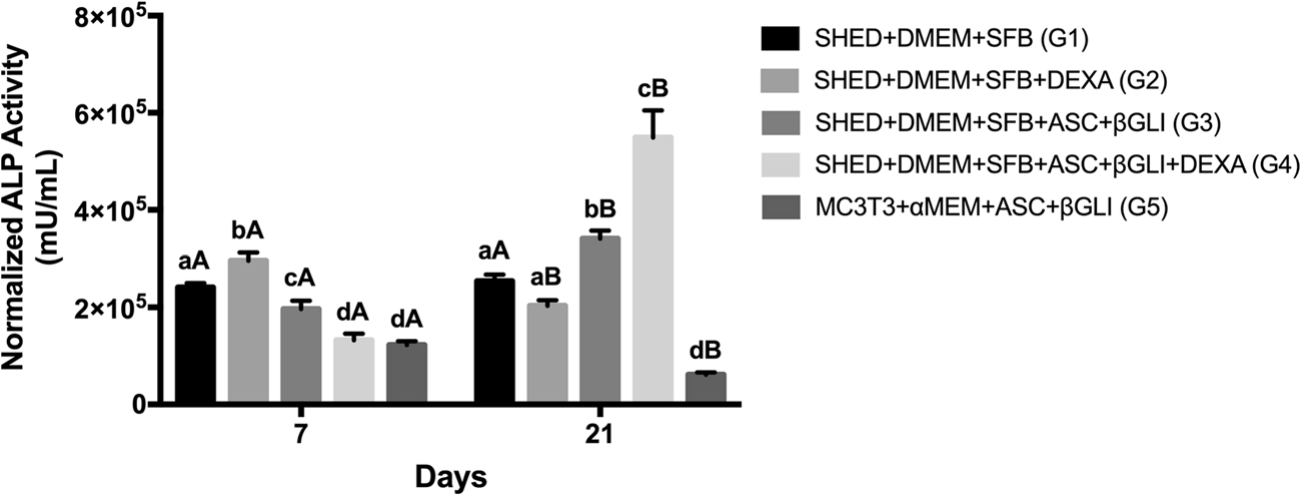
Alkaline phosphatase (ALP) activity by ALP Assay Kit Fluorimetric on days 7 (*n* = 12 for each group) and 21 (*n* = 12 for each group). ALP quantification results were normalized to the DNA content. Different lower-case letters refer to a significant difference (ANOVA/Tukey test, *p* < 0.05) among groups at the same experimental time. Different capital letters indicate significant differences (Student test, *p* < 0.05) between the experimental times for the same group

Regarding the ALP activity, Fig. 2 demonstrates that G2 presented the highest enzymatic activity followed by G1, G3, G4, and G5 on day 7 (*p* < 0.001). On day 21, G4 showed the highest ALP activity followed by G3, while G5 demonstrated the lowest values (*p* < 0.0001). Therefore, G4 promoted less ALP activity than G3 on day 7, while the inverse occurred on day 21. A reduction in the ALP activity from day 7 to 21 was observed for G2 (*p* = 0.0001) and G5 (*p* < 0.0001), while an increase was perceived for G3 (*p* < 0.0001) and G4 (*p* = 0.0004).

### Calcium quantification in the extracellular medium

**Fig. 3:**
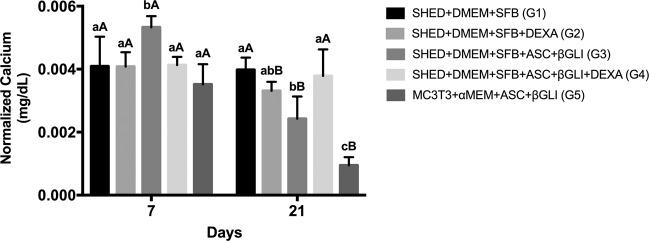
Free calcium quantification by the QuantiChromTM Calcium Assay Kit in the extracellular media on days 7 (*n* = 12 for each group) and 21 (*n* = 12 for each group). Calcium quantification results were normalized to the DNA content. Different lower-case letters refer to a significant difference (ANOVA/Tukey test, *p* < 0.05) among groups at the same experimental time. Different capital letters indicate significant differences (Student test, *p* < 0.05) between the experimental times for the same group

According to Fig. 3, G3 presented the highest free calcium quantification on day 7 (*p* < 0.0001), while G5 showed the lowest amount on day 21 (*p* < 0.0001). On day 21, G4 demonstrated more free calcium quantification than G3 (*p* = 0.0021). Also, a reduction of calcium in the extracellular media for G2 (*p* = 0.0017), G3 (*p* < 0.0001), and G5 (*p* < 0.0001) from day 7 to 21 was observed.

### Extracellular matrix mineralization

**Fig. 4:**
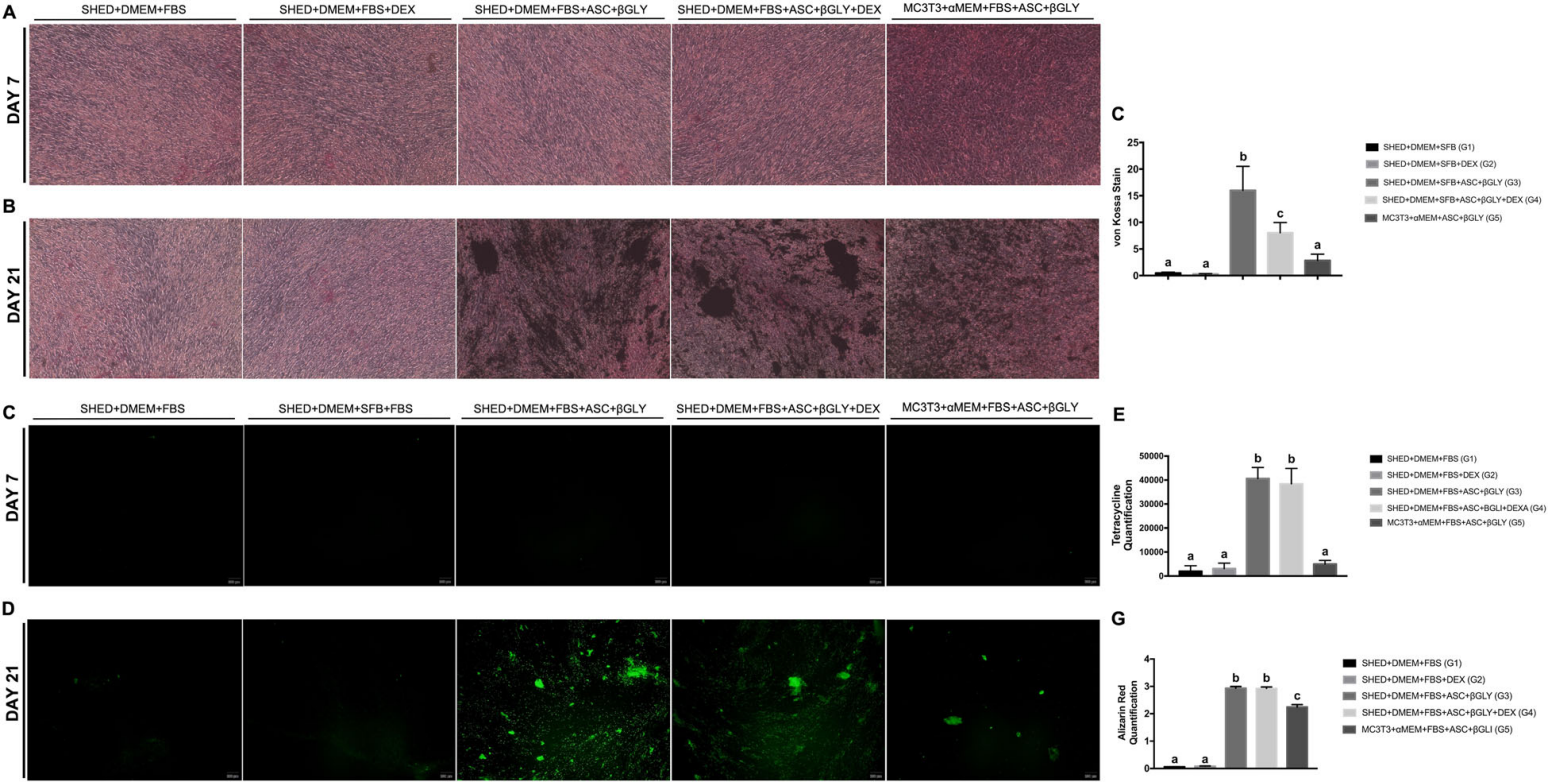
Extracellular matrix mineralization. **A** Von kossa staining on day 7 (*n* = 12 for each group) and **B** on day 21 (*n* = 12 for each group); **C** quantification of extracellular matrix mineralization by te Von kossa assay on day 21 using Image J software (NIH) (*n* = 12 for each group). Different lower-case letters refer to a significant difference (ANOVA/Tukey test, *p* < 0.05) among groups at the same experimental time; **D** tetracycline fluorescence on day 7 (*n* = 12 for each group) and **E** on day 21 (*n* = 12 for each group); **F** quantification of mineralization nodules by the tetracycline assay on day 21 (*n* = 12 for each group). Different lower-case letters refer to a significant difference (ANOVA/Tukey test, *p* < 0.05) among groups at the same experimental time; **G** red alizarin quantification on day 21 (*n* = 12 for each group). Different lower-case letters refer to a significant difference (ANOVA/Tukey test, *p* < 0.05) among groups at the same experimental time

Von Kossa staining results revealed high cell densities in all groups on day 7, but the absence of mineralization (Fig. 4A). On day 21, all groups were able to mineralize extracellular matrix (Fig. 4B). In addition, G3 revealed the highest mineralization levels (*p* < 0.0001), followed by G4 and G5 (Fig. 4C). Regarding the extracellular matrix mineralization quantification by tetracycline assay, a very tinny quantification of tetracycline (Fig. 4D) occurred on day 7. However, a sharp increase in tetracycline quantifications for G3, G4, and G5 occurred on day 21 (Fig. 4E, F), with no significant difference between G3 and G4 (*p* = 0.9243). According to Fig. 4G, alizarin red staining corroborates the results of tetracycline quantification, indicating no significant difference between G3 and G4 (*p* = 0.9998).

## Discussion

Despite DEX has been widely used to induce osteogenesis in vitro [23], different effects have been related to this glucocorticoid on MSCs and osteoblasts [12–15]. Therefore, due to the inconsistency regarding the application of DEX as supplementation of osteogenic media and the importance of using a reliable positive control group, the purpose of this study was to analyze in vitro the influence of DEX media supplementation on the DNA content and differentiation of SHED into an osteoblastic-like lineage. In summary, we observed that the osteogenic media supplemented with ASC and β-GLY demonstrated similar effects on SHED in the presence or absence of DEX for the DNA content assay (day 21) and the capacity to mineralize the extracellular matrix according to tetracycline and alizarin red quantifications (day 21). In addition, the presence of DEX in the osteogenic medium promoted less ALP activity (day 7) and mineralization of extracellular matrix according to von Kossa staining (day 21), and more free calcium quantification at extracellular medium (day 21). On the other hand, the supplementation with DEX without ASC and β-GLY did not demonstrate the capacity to mineralize the extracellular matrix, with similar effects of culture media with FBS only. These findings are relevant since they describe the role of DEX in the osteogenic culture media supplementation for SHED, emphasizing the terminal phase of differentiation, i.e., the deposition of the mineralized matrix by the differentiated cells. Thereby, the present study can be useful to assist in the choice of the osteogenic supplementation when SHED is used as a positive control in the osteogenic differentiation assays. In addition, it is pertinent to notice that SHED exhibit distinct biological characteristics compared to other dental MSCs [3, 5–7].

As previously mentioned, DEX impact over MSCs is related to the treatment duration, concentration, and stage of osteoblastic differentiation [12, 13]. Herein, we have used the same concentrations of osteogenic substances as previously reported [13, 18, 24]. Nevertheless, distinct concentrations have been reported in the literature, treating cells with 50-μg/mL ASC, 5-mM β-GLY, and 100-nM DEX, and [25], or 50-mg/L ASC, 10-mmol/L βGLY, and 0.1-µmol/L DEX [26]. Therefore, the concentration of β-GLY from these two studies was different from that employed in our research, while there was no difference in ASC or DEX concentrations, although the units of measurement differed. Low doses of DEX, such as the dose applied herein, favored MSCs proliferation and angiogenesis in vitro and protects against apoptosis [10].

It is relevant to discuss that we have proposed a protocol by supplementing culture media with tetracycline, which is not deleterious to the cells, has the capacity to bind the mineral deposits and is fluorescent [6, 27]. It is well known that tetracycline can be incorporated into a mineralizing dentin matrix, which may result in the staining of forming teeth when systemically administered [28]. Previous in vivo studies [6, 27] used injections of tetracycline hydrochloride to demonstrate calcium mineral deposition through fluorescence. It is relevant to mention that the proposed protocol using tetracycline does not require cell fixation before staining, as required by the von Kossa and alizarin red assays [29, 30]. Therefore, the continuous observation of dynamic changes during the formation of mineralized nodules in living cell cultures becomes possible, with similar abilities to specifically label mineral deposition and avoid the deleterious effects of cell fixation, as previously reported [31].

According to the cellular metabolism, undifferentiated cells, at their initial stages, proliferate rapidly to achieve a sufficient cell density before starting the differentiation process under specific stimuli. When cells receive the signals to differentiate, they become committed and gradually stop proliferating while starting the expression of early markers, such as ALP and osterix. Throughout the differentiation process, cells express markers, such as collagen type I, osteopontin, osteonectin, and osteocalcin. This sequential upregulation leads to the differentiation of MSCs into osteoblast-like cells capable to deposit minerals in the extracellular matrix [12, 32, 33]. Herein, the reduction on SHED DNA content from day 7 to day 21 for both groups that received the osteogenic medium, regardless of the absence or presence of DEX (G3 and G4, respectively), probably due to the commitment of these cells to the osteogenic differentiation process was noted [12, 32, 33]. In addition, we observed that SHED treated with ASC and β-GLY in the presence of DEX (G4) promoted more DNA content compared to cells treated only with ASC and β-GLY (G3) at day 7, which agrees with Beloti and Rosa [34]. On the other hand, Chadipiralla et al. [13] observed that SHED treated with osteogenic medium including DEX demonstrated less proliferation rates compared to SHED treated without DEX. Regarding the DNA content, it is important to mention that besides the reduction in cell proliferation during cell commitment to differentiation, we could observe that some cells detached the bottom of the plates while the extracellular matrix was mineralized. In addition, differentiated cells are harder to be removed from the plastic through enzymatic procedures for the experiment and it can also explain the reduction in DNA content herein observed.

Interestingly, the SHED group that received ASC, β-GLY, and DEX (G4) presented an increase on ALP activity at day 21, probably due to the later stage on the differentiation process of these cells, since, at this later experimental time (day 21), the osteoblast committed cells should be mineralizing the extracellular matrix and not be dedicated to ALP function. Moreover, at the initial time (day 7), the SHED group that received ASC, β-GLY, and DEX (G4) demonstrated fewer ALP activity than SHED treated without DEX (G3), according to Chadipiralla et al. [13]. This finding indicates that the DEX addition to the osteogenic medium did not seem to improve the osteogenic differentiation of SHED. Conversely, Beloti and Rosa [34] demonstrated a positive effect of DEX in ALP activity. We also observed that the regular medium (G1) promoted more ALP activity than the osteogenic medium with DEX (G4). This finding is divergent from Loebel et al., Klontzas and Tsiridis, and Miao et al. [24–26], who evaluated human bone marrow stem cells, umbilical cord blood MSCs, and cells from fresh human skeletal muscle tissue, respectively.

Another interesting finding of the present study is the amount of calcium quantified in the supernatant. At day 7 of the experiments, most groups presented similar concentrations of Ca^2+^, except G3 that showed the highest calcium availability. As expected, there was a sharp reduction in free calcium quantification at day 21, since the calcium availability in the supernatant decreased as cell differentiation arises and free calcium is trapped for mineral matrix formation, as observed for the positive control group (G5). This finding is corroborated by the tetracycline and alizarin red quantitative extracellular matrix mineralization assays [31]. In this sense, on day 21, the DEX addition to the osteogenic medium was supposed to decrease the free calcium quantification. However, the opposite was observed, since G4 (osteogenic medium with DEX) demonstrated more free calcium than G3 (osteogenic medium without DEX). In addition, according to tetracycline and alizarin red quantification, the supplementation with DEX into the osteogenic medium (G4) did not increase the extracellular matrix mineralization. Consequently, the DEX addition to the osteogenic medium did not seem to improve the SHED differentiation into an osteoblast-like cell capable to mineralize the extracellular matrix. Some studies are in accordance with our findings and reported a downstream of bone matrix formation as osteoblastic differentiation progresses under DEX treatment [12–15, 35]. Chadipiralla et al. [13] previously demonstrated that DEX addition into the osteogenic medium did not improve the osteoblast differentiation of SHED. Rimando et al. [12] explained these findings by the GR that is directly targeted by early (Runx2) and late (osteocalcin and bone sialoprotein) bone markers in osteoblasts. GR positively regulates Runx2 while inhibits osteocalcin transcriptions. Over MC3T3-E1 cells, DEX also led to the inhibition of osteoblastic differentiation through the downregulation of osteocalcin gene expression [14]. Furthermore, MSCs under DEX supplementation were found to be more likely to differentiate into an adipocyte rather than osteoblast lineage [16]. Conversely to these findings, Beloti and Rosa [34] verified that supplementation with DEX into osteogenic media promoted more extracellular matrix mineralization through the alizarin red assay. Klontzas and Tsidiris [24] showed that the association of DEX, ASC, and β-GLY induced more mineralization of the extracellular matrix compared to the non-osteogenic medium. In addition, we perceived that the supplementation with DEX without ASC and β-GLY (G2) was able to induce a minute mineralization of the extracellular matrix, with similar effects of culture media with FBS only. These divergent findings regarding DEX use could be explained by the positive stimulus of osteoblast lineage commitment in the presence of DEX at the early stages of differentiation and the negative influence over bone matrix formation as osteoblastic differentiation progresses [12]. In addition, the fact that MSCs from different sources exhibit distinct biological properties [3, 5–7] could also explain the divergences among some reports and findings observed herein. It is relevant to mention that our findings are in agreement with Chadipiralla et al. [13] who also evaluated SHED treated with the same DEX concentration and compatible time points described here.

Since some studies have shown beneficial effects of DEX in the early stages, but negative effects in the terminal phases of the osteoblast differentiation of MSCs through gene expression of bone-specific markers [12], our study focused in the terminal phase of differentiation, evaluating the differentiated cells capable to deposit mineralized matrix. Therefore, we evaluated the free calcium at the extracellular medium and the mineralization of extracellular matrix by alizarin red, von Kossa, and tetracycline staining. Nevertheless, it was considered important to assess DNA content and ALP activity since DEX has been shown to have a positive impact on the initial phases of osteoblastic differentiation. Moreover, our findings corroborate that the use of DEX should be regulated over the differentiation process or even suppressed since the bone matrix formation might be enhanced while minimizing the adverse effects of continuous DEX exposure [12, 13]. However, it is important to mention that different MSC have been demonstrated distinct biological characteristics [3, 5–7].

## Conclusion

Based on the performed experiments, it can be concluded that the presence of DEX in the osteogenic media supplementation did not interfere with SHED commitment into mineral matrix depositor cells. For this reason, we suggest that DEX may be omitted from culture media supplementation for SHED differentiation in in vitro studies. Therefore, osteogenic media composed by DMEM with 10% FBS, 50-µM L-ascorbic acid, and 10-mM βGLY seem to be sufficient for osteogenic differentiation of SHED. Further studies are desired to observe the role of DEX in the osteogenic media supplementation in MSCs from other dental sources. In addition, the mechanisms by which DEX acts in the cell culture media are still unknown and require further studies.

## References

[1] Li Z, Jiang C, An S, Cheng Q, Huang Y, Wang Y (2014). Immunomodulatory properties of dental tissue-derived mesenchymal stem cells. Oral Dis.

[2] Bakopoulou A, About I (2016). Stem cells of dental origin: current research trends and key milestones towards clinical application. Stem Cells Int.

[3] Wang H, Zhong QI, Yang T, Qi Y, Fu M, Yang XI (2018). Comparative characterization of SHED and DPSCs during extended cultivation in vitro. Mol Med Rep.

[4] Liu J, Yu F, Jiang B, Zhang W, Yang J, Xu G-T (2015). Characteristics and potential applications of human dental tissue-derived mesenchymal stem cells. Stem Cells.

[5] Miura M, Gronthos S, Zhao M, Lu B, Fisher LW, Robey PG (2003). SHED: stem cells from human exfoliated deciduous teeth. Proc Natl Acad Sci U S A.

[6] Sakai VT, Zhang Z, Dong Z, Neiva KG, Machado MAAM, Shi S (2010). SHED differentiate into functional odontoblasts and endothelium. J Dent Res.

[7] Winning L, Karim IA El, Lundy FT. A comparative analysis of the osteogenic potential of dental mesenchymal stem cells. Stem Cells Dev. 2019;28:1050–8. 10.1089/scd.2019.0023 31169063

[8] Mushtaq T, Farquharson C, Seawright E, Ahmed SF (2002). Glucocorticoid effects on chondrogenesis, differentiation and apoptosis in the murine ATDC5 chondrocyte cell line. J Endocrinol.

[9] Drake AJ, Walker BR (2004). The intergenerational effects of fetal programming: non-genomic mechanisms for the inheritance of low birth weight and cardiovascular risk. J Endocrinol.

[10] Ishida Y, Heersche JNM (1998). Glucocorticoid-induced osteoporosis: both in vivo and in vitro concentrations of glucocorticoids higher than physiological levels attenuate osteoblast differentiation. J Bone Min Res.

[11] Ito S, Suzuki N, Kato S, Takahashi T, Takagi M (2007). Glucocorticoids induce the differentiation of a mesenchymal progenitor cell line, ROB-C26 into adipocytes and osteoblasts, but fail to induce terminal osteoblast differentiation. Bone.

[12] Rimando M, Wu HH, Liu YA, Lee CW, Kuo SW, Lo YP (2016). Glucocorticoid receptor and Histone deacetylase 6 mediate the differential effect of dexamethasone during osteogenesis of mesenchymal stromal cells (MSCs). Sci Rep.

[13] Chadipiralla K, Yochim JM, Bahuleyan B, Huang C-YC, Garcia-Godoy F, Murray PE (2010). Osteogenic differentiation of stem cells derived from human periodontal ligaments and pulp of human exfoliated deciduous teeth. Cell Tissue Res.

[14] Lian JB, Shalhoub V, Aslam F, Frenkel B, Green J, Hamrah M (1997). Species-specific glucocorticoid and 1,25-dihydroxyvitamin D responsiveness in mouse MC3T3-E1 osteoblasts: dexamethasone inhibits osteoblast differentiation and vitamin D down-regulates osteocalcin gene expression. Endocrinology.

[15] Wang H, Pang B, Li Y, Zhu D, Pang T, Liu Y (2012). Dexamethasone has variable effects on mesenchymal stromal cells. Cytotherapy.

[16] Li J, Zhang N, Huang X, Xu J, Fernandes JC, Dai K (2013). Dexamethasone shifts bone marrow stromal cells from osteoblasts to adipocytes by C/EBPalpha promoter methylation. Cell Death Dis.

[17] Oliveira NK, Salles THC, Pedroni AC, Miguita L, D’Ávila MA, Marques MM (2019). Osteogenic potential of human dental pulp stem cells cultured onto poly-e-caprolactone/poly (rotaxane) scaffolds. Dent Mater Acad Dent Mater.

[18] Zuk PA, Zhu M, Mizuno H, Huang J, Futrell W, Katz AJ (2001). Multilineage cells from human adipose tissue: implications for cell-based therapies. Tissue Eng.

[19] Cruz ACC, Cardozo FTG, de S, Magini RDS, Simões CMO (2019). Retinoic acid increases the effect of bone morphogenetic protein type 2 on osteogenic differentiation of human adipose-derived stem cells. J Appl Oral Sci.

[20] Callegari-Jacques SM. Bioestatística. Princípios e aplicações. Porto Alegre: Artmed; 2003.

[21] Cho Y, Hong J, Ryoo H, Kim D, Park J, Han J (2015). Osteogenic responses to zirconia with hydroxyapatite coating by aerosol deposition. J Dent Res.

[22] Pang Y, Yuan X, Guo J, Wang X, Yang M, Zhu J (2019). The effect of liraglutide on the proliferation, migration, and osteogenic differentiation of human periodontal ligament cells. J Periodontal Res.

[23] Heng BC, Cao T, Stanton LW, Robson P, Olsen B (2004). Strategies for directing the differentiation of stem cells into the osteogenic lineage in vitro. J Bone Min Res.

[24] Klontzas ME, Tsiridis E (2017). Metabolomics analysis of the osteogenic differentiation of umbilical cord blood mesenchymal stem cells reveals. Stem Cells Dev.

[25] Loebel C, Czekanska EM, Bruderer M, Salzmann G, Alini M, Stoddart MJ (2015). In vitro osteogenic potential of human mesenchymal reverse primer. Tissue Eng.

[26] Miao C, Zhou L, Tian L, Zhang Y, Zhang W, Yang F (2017). Osteogenic differentiation capacity of in vitro cultured human skeletal muscle for expedited bone tissue engineering. BioMed Res Int.

[27] Kawasaki K, Tanaka S, Ishikawa T (1977). On the incremental lines in human dentine as revealed by tetracycline labelling. J Anat.

[28] Ciarlone AE, Johnson RD, Pashley DH (1989). Further characterization of tetracycline’s quantitative binding to dentin. J Endod.

[29] Puchtler H, Meloan SN, Terry MS (1969). On the history and mechanism of alizarin red S stains for calcium. J Histochem Cytochem.

[30] Bills CE, Eisenberg H, Pallante SL (1974). Complexes of organic acids with calcium phosphate: the Von Kossa stain as a clue to the composition of bone mineral. Johns Hopkins Med J.

[31] Wang Y, Liu Y, Maye P, Rowe DW (2006). Examination of mineralized nodule formation in living osteoblastic cultures using fluorescent dyes. Biotechnol Prog.

[32] Green E, Todd B, Heath D (1990). Mechanism of glucocorticoid regulation of alkaline phosphatase gene expression in osteoblast-like cells. Eur J Biochem.

[33] Franceschi RT, Xiao G (2003). Regulation of the osteoblast-specific transcription factor, Runx2: responsiveness to multiple signal transduction pathways. J Cell Biochem.

[34] Beloti MM, Rosa AL (2005). Osteoblast differentiation of human bone marrow cells under continuous and discontinuous treatment with dexamethasone. Braz Dent J.

[35] Malladi P, Xu Y, Yang GP, Longaker MT (2006). Functions of vitamin D, retinoic acid, and dexamethasone in mouse adipose-derived mesenchymal cells. Tissue Eng.

